# Using sea urchin gametes and zygotes to investigate centrosome duplication

**DOI:** 10.1186/s13630-016-0043-3

**Published:** 2016-09-06

**Authors:** Greenfield Sluder

**Affiliations:** Department of Cell and Developmental Biology, University of Massachusetts Medical School, S6-212, 55 Lake Avenue North, Worcester, MA 01655 USA

**Keywords:** Centriole, Centrosome, Duplication, Echinoderm, Egg, Sea urchin, Sperm, Zygote

## Abstract

Centriole structure and function in the sea urchin zygote parallel those in mammalian somatic cells. Here, I briefly introduce the properties and attributes of the sea urchin system that make it an attractive platform for the study of centrosome and centriole duplication. These attributes apply to all echinoderms readily available from commercial suppliers: sea urchins, sand dollars, and starfish. I list some of the practical aspects of the system that make it a cost- and time-effective system for experimental work and then list properties that are a “tool kit” that can be used to conduct studies that would not be practical, or in some cases not possible, with mammalian somatic cells. Since centrioles organize and localize the pericentriolar material that nucleates the astral arrays of microtubules (Bobinnec et al. in J Cell Biol 143(6):1575–1589, 1998), the pattern of aster duplication over several cell cycles can be used as a reliable measure for centriole duplication (Sluder and Rieder in J Cell Biol 100(3):887–896, 1985). Descriptions of the methods my laboratory has used to handle and image echinoderm zygotes are reviewed in Sluder et al. (Methods Cell Biol 61:439–472, 1999). Also included is a bibliography of papers that describe additional methods.

## Background

### Sperm and egg

**Fig. 1:**
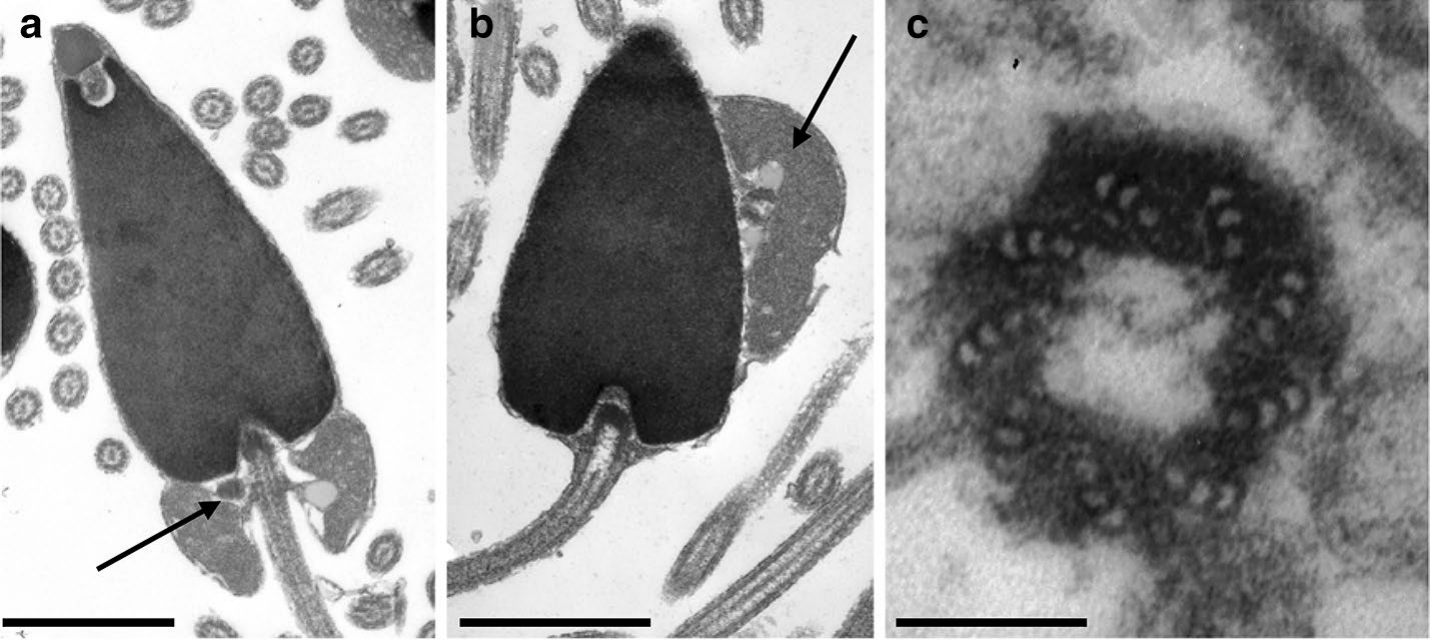
Ultrastructure of sea urchin sperm basal bodies and distal centrioles. **a** Longitudinal section of a sperm head. At the base of the head one can see the basal body—flagellar apparatus, the mitochondrion, and the short distal centriole lying between the mitochondrion and the nucleus. Here, the distal centriole in tangential section appears as an electron-dense patch to the *left* of the basal body (*arrow*). *Scale bar* 1 μm. **b** Longitudinal section of the head of a sperm treated with gluconate-glycine buffer. The dense cap on the proximal end of the basal body is located where it is mechanically attached to the hof in the base of the nucleus. The mitochondrion has moved to the side of the nucleus along with the distal centriole seen in longitudinal section (*arrow*). *Scale bar* 1 μm. **c** Cross-section of an isolated distal centriole. *Scale bar* 0.1 μm

The sea urchin sperm contains two centrioles. One serves as the basal body of the flagellum and has a prominent electron-dense cap on the proximal end where it is firmly anchored in a hof at the base of the sperm nucleus (Fig. 1a, b). In cross-section the basal body has a canonical nine-triplet structure. The second centriole, located between the mitochondrion and the nucleus (Fig. 1a, b), also has a canonical nine-triplet structure that is embedded in an electron-dense, ring-shaped matrix (Fig. 1c). This distal centriole in the nomenclature of [4] is short with an aspect ratio reminiscent of a “Life Saver” candy.

When a female sea urchin is induced to spawn, the eggs are shed in a post-meiotic state with a haploid interphase nucleus. The unfertilized egg is believed to be devoid of centrioles as none have been reported from ultrastructural studies. When the cell cycle is activated in unfertilized eggs, there is no activity that organizes a duplicating centrosome and no centrioles are assembled [5]. Centrosome inheritance in echinoderms is paternal; both centrioles in the sperm are contributed to the zygote and after duplication they organize the centrosomes used in development.

### Zygote

**Fig. 2:**
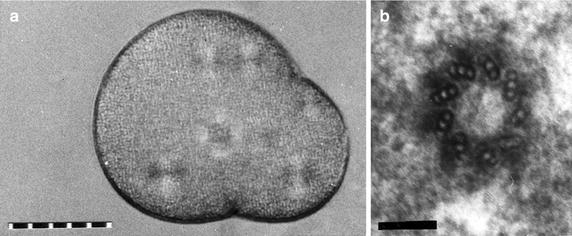
**a** Enucleated sea urchin zygote followed in vivo to characterize centrosome duplication. Each duplicated centrosome organizes a birefringent aster as visualized with a polarization microscope. The birefringence and appearance of the asters indicate that this enucleated zygote was in mitosis when the photograph was taken. The refractile sphere, slightly out of focus in the center of the cell, is a drop of the mineral oil used to cap the micropipette employed to enucleate the zygote. Scale divisions are 10 μm apart. **b** This particular zygote was recovered from the preparation, fixed, and serial semi-thick sectioned. Shown is a cross-section of a centriole in one of the centrosomes. The other centriole was found in a different section. *Scale bar* 0.1 μm

Within approximately 15–30 min after fertilization, the two sperm centrioles have organized a sperm aster and duplicate at this time of DNA synthesis [6]. The female pronucleus moves toward the focus of the sperm aster and fuses with the male pronucleus to form the zygote nucleus. Later the mother–daughter centriole pairs organize asters that separate around the zygote nucleus prior to first mitosis. During the first embryonic mitoses, all centrioles have a canonical nine-triplet structure (Fig. 2b) and sometimes appear to be slightly separate within a cloud of fibro-granular pericentriolar material during mitosis (see serial section series in [2, 7]). During the early zygote mitoses, there is no indication that the pericentriolar material preferentially accumulates around the older or mother centriole, as is the case for mammalian somatic cells. During mitosis, the mother–daughter centrioles do not always exhibit a tight orthogonal arrangement which raises questions about the extent to which they are mechanically and functionally engaged at this point in the cell cycle. In telophase, the centrosome flattens and DNA synthesis promptly begins—even before all the karyomeres (individual chromosomes or groups of chromosomes around which nuclear envelopes have assembled) have fused to form an interphase nucleus [8]. The centrioles duplicate at this time [9].

### Practical considerations

1. Sea urchins and other echinoderms are a cost-effective experimental system. Large quantities of eggs and sperm can be collected from one or a few organisms for organelle isolation and biochemical studies. The gametes and zygotes live in sea water; they function under atmospheric conditions; and they do not require sterile conditions. One must culture the zygotes at a temperature similar to that at which that particular species lives—typically this is below normal room temperatures.
2. The eggs are large (~70–140 μm in diameter depending on the organism). During the early mitoses the spindles and asters are large compared to somatic cells. The eggs of some species are optically clear and thus well suited to in vivo imaging using polarization or differential interference contrast optics. Importantly, one can follow the behavior of spindles and centrosomes in vivo without fluorescent probes using a polarization microscope (Figs. 2a, 3). Since centrioles organize the pericentriolar material that nucleates the asters, the pattern of aster doubling can be used as a measure of the number and duplication of the centrioles. For example, a centrosome containing the normal two centrioles precisely doubles from one cell cycle to the next. A centrosome containing one centriole does not double in the first cell cycle but then doubles between all subsequent cycles (see [2]).

**Fig. 3:**
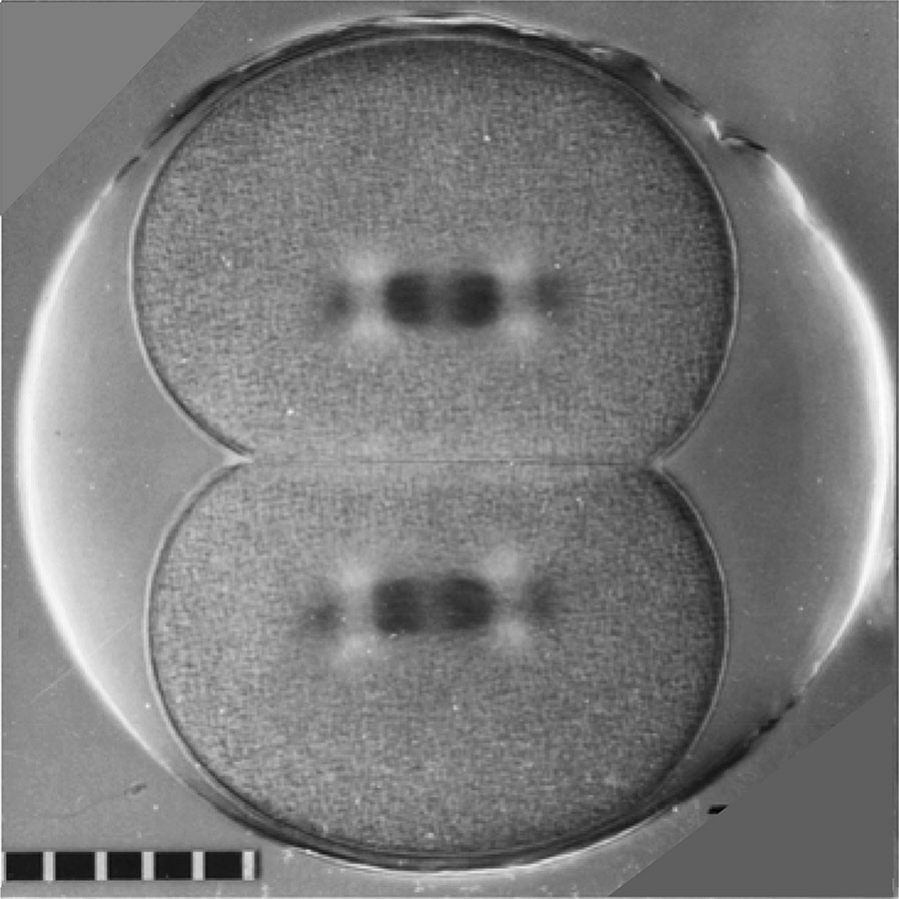
Second mitosis in a *Lytechinus pictus* zygote. This image shows use of the polarizing microscope to image spindles and asters in living zygotes of a sea urchin that has optically clear eggs. The spindles are negatively compensated and appear dark. Quadrants of the asters are bright because the microtubules in those quadrants are oriented at right angles to those of the central spindle. Scale divisions are 10 μm apart
3. One can follow individual zygotes in vivo and then recover each for correlative fixed cell light microscopy or electron microscopy (Fig. 2a, b). To precisely characterize the number and spatial arrangement of centrioles in zygotes, one needs to use serial semi-thick section analysis (see [10]). A method for recovering and fixing for electron microscopy individual zygotes previously followed in vivo in microinjection preparations is described in [7]. For specifics on sealed or open-faced imaging preparations for microinjection/micromanipulation see [3].
4. The zygote cell cycle is rapid and roughly synchronous for a population of eggs fertilized at the same time. For the commonly used echinoderms with native temperatures ranging from 13 to 22 ℃, cell cycle duration ranges from 40 min to 1.5 h depending upon the temperature. Since the investigation of centrosome/centriole duplication often requires following the cells for multiple cell cycles, echinoderm zygotes allow one to do an experiment in a morning or afternoon, not the several days needed for somatic cells. Since experiments usually last a few hours, the investigator can be continuously present at the microscope to follow/image in detail 5 or more zygotes per run. For the use of polarization or differential interference optics, a rotating stage allows one to align each zygote to optimize contrast. Simple time lapse microscope systems, without computer programmed focus and stage position, allow one to follow only a few cells at a time, often without proper orientation in the field.
5. The sea urchin zygote is robust and will tolerate experimental insults that would arrest kill untransformed somatic cells. Zygotes can be mechanically fragmented and better survive micromanipulation than cultured cells. Also, zygotes have a finite but much greater tolerance for exposure to blue and near UV light than untransformed somatic cells. For example, exposure of untransformed human cells (RPE1) to 488 nm light delivered through a conventional epifluorescence pathway leads to dose-dependent cell cycle arrest or cell death (Douthwright and Sluder, submitted). Sea urchin zygotes tolerate equivalent exposures to 366 nm light even though it is more energetic. In this regard, a note of caution: when imaging Hoechst-stained metaphase sea urchin chromosomes in the DAPI channel of a fluorescence microscope, one must be careful to limit exposures to the excitation light. Longer exposures can cause the sister chromosomes to stick together and when the cell enters anaphase, there is chromosome bridging and non-disjunction.
6. Even though eggs and zygotes are not amenable to transfection, there are well-established methods for microinjecting eggs and zygotes prior to following their behavior in vivo.
7. The genomes of *S. purpuratus* and *L. variegatus* have been sequenced giving the investigator access to the specific DNA sequences of centriolar proteins. New expression of specific proteins in zygotes can be inhibited by the injection of engineered antisense Morpholino oligonucleotides (reviewed in [11]). Recently, genome editing in individual sea urchin zygotes with the CRISPR/Cas9 system has been reported [12].

## Tool kit

Listed below are some of the characteristics of the sea urchin zygote that have proven useful tools in the study of centrosome duplication.

**Fig. 4:**
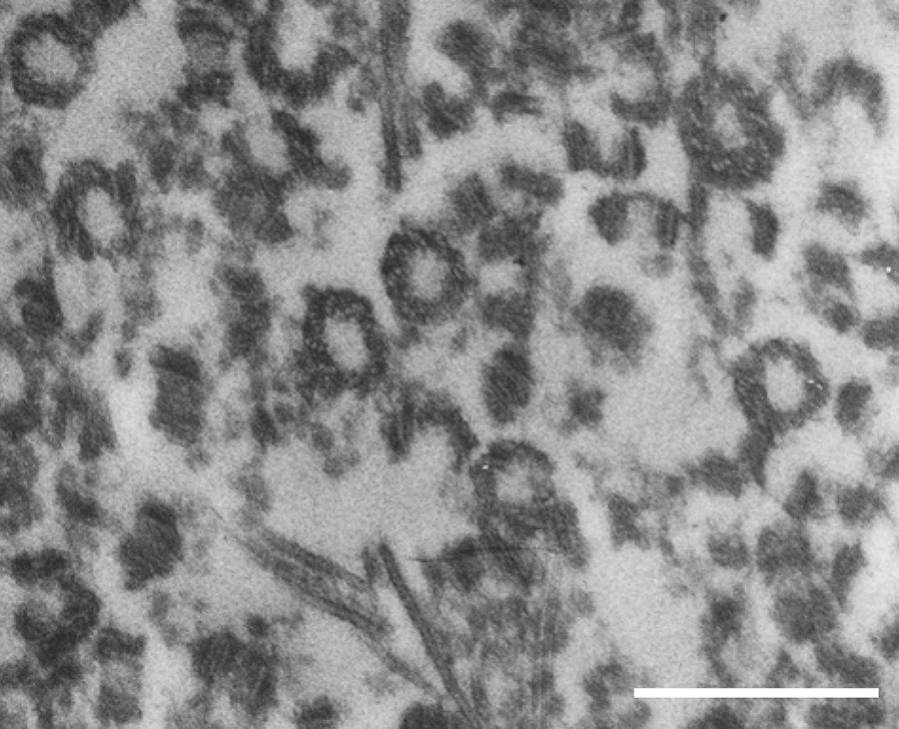
Electron micrograph of a section through a pellet of distal centrioles isolated from sea urchin sperm. *Scale bar* 0.5 μm

1. Centrosome duplication and cell cycle progression do not depend upon transcription. When the zygote nucleus is removed, centrosome/centriole duplication repeatedly occurs in proper coordination with cycles of astral microtubule assembly/disassembly and attempts at cleavage [7].
2. Even before fertilization the sea urchin egg contains abundant pools of all subunits needed to support repeated centrosome duplication to make many centrosomes. Centrosome/centriole duplication proceeds over at least several hours in zygotes when protein synthesis is completely blocked [13]. The presence of relatively stable centriole/centrosome subunit pools may limit the usefulness of Morpholino oligonucleotide and the CRISPR/Cas9 approaches to diminish specific centrosomal protein levels, at least in early development.
3. Centrosomes repeatedly duplicate when the zygote is arrested in S phase [14]. For *Lytechinus pictus*, the period of reduplication is on average 148 min (range 40–257 min) [15].
4. Zygotes can be fragmented by passing them through a plastic screen shortly after the fertilization envelope is removed. Since this is done before pronuclear fusion, one obtains a mixture of viable cycling enucleated cell fragments; fragments that contain just a male or a female pronucleus or fragments with both pronuclei [5]. The centrosome(s) remain associated with the male pronucleus.
5. The cell cycle of unfertilized eggs can be artificially activated by a variety of agents. One of the easiest and most controllable methods is to transiently raise their internal pH with ammoniated sea water [16]. Such activated eggs, or experimentally produced fragments of fertilized eggs that contain only the female pronucleus, organize at mitosis a single radial array of microtubules (a monaster) at the site of the female pronucleus. However, this monaster does not duplicate and becomes progressively less well organized from one mitosis to the next [5]. At the ultrastructural level, the monaster consists of a hollow fenestrated sphere of electron-dense material from which microtubules emanate outward. Serial section analysis revealed that there are no morphological centrioles present [5]. This system could be used to study the properties and composition of pericentriolar material free from centrioles.
6. Parthenogenesis: Starting in the late 1800s, methods were described to induce the de novo formation of centrosomes in artificially activated, unfertilized eggs leading to parthenogenetic development. In short, the activated egg is transiently dehydrated with hypertonic sea water, or sea water containing glucose or D_2_O during the first cell cycle. The centrosomes that assemble (called cytasters) contain centrioles [17]. With carefully modulated treatments, one or two centrosomes assemble in each egg and support normal early development of haploid or diploid embryos ([18]; early work reviewed in [19]). With stronger treatments, many cytasters per egg are assembled, some with multiple centrioles [17, 20]. Fertilization can also be used to activate the egg and dehydrating conditions will induce the assembly of supernumerary cytasters that duplicate at each cell cycle (Sluder and Lewis, unpublished). The rapid cell cycle and the ability to have large quantities of roughly synchronous eggs may offer advantages for the use of modern methods to study of this poorly understood phenomenon that also occurs in transformed and untransformed human cells [21, 22].

7. Sea urchin sperms offer a favorable system for centriole isolation given that nature has removed almost all the cytoplasm from these cells. One can collect large quantities of sperm and detergent lysis in a gluconate-glycine buffer (see [3]) allows one to collect the short distal centrioles (Fig. 4) from the supernatant, albeit with some axoneme and mitochondrial remnant contamination (also see [23]). When these isolated distal centrioles are microinjected into fertilized sea urchin eggs, they organize asters in a dose-dependent fashion (Sluder unpublished). One can also pellet the sperm nuclei with the firmly attached basal body—flagellar apparatus.

## Basal bodies

The sea urchin system can also be used to investigate basal body biology. Centrioles function as basal bodies in the sperm and in development starting at the ciliated blastula stage. Work to date on basal bodies in this system has been largely limited to conventional thin-section ultrastructural characterizations. Nevertheless, the sea urchin embryo provides what may be a unique vehicle to study the repetitive inter-conversion of centrioles to basal bodies [24]. In early development, the embryo becomes a hollow ball of cells—the blastula. Shortly before the embryo hatches from the fertilization envelope, each cell assembles a single motile cilium. Interestingly, the cells are still proliferating. After mitosis, the mother–daughter centriole pair moves to the apical cortex of the cell and the older or mother centriole acts as a basal body to organize the assembly of the ciliary axoneme. While in this configuration the centrioles duplicate and shortly before entry into mitosis, the axoneme is rapidly pulled back into the cell and detaches from the mother centriole. During mitosis, the two mother/daughter centriole pairs organize the poles of the mitotic spindle. This sequence of events is repeated in the next cell cycle.

## Conclusions

Sea urchin gametes and zygotes have proven to be a useful experimental system from which much basic information on centrosome duplication has been developed. Some of the important studies would not have been practical or indeed possible with cultured cells. Although the use of this system in centrosome biology has not been much used in recent years, its several practical advantages make it wide open for new and imaginative approaches with the modern tools of molecular biology.
